# Global Future Drought Layers Based on Downscaled CMIP6 Models and Multiple Socioeconomic Pathways

**DOI:** 10.1038/s41597-025-04612-w

**Published:** 2025-02-19

**Authors:** Diogo S. A. Araujo, Brian J. Enquist, Amy E. Frazier, Cory Merow, Patrick R. Roehrdanz, Gabriel M. Moulatlet, Alex Zvoleff, Lei Song, Brian Maitner, Efthymios I. Nikolopoulos

**Affiliations:** 1 Department of Civil and Environmental Engineering, Rutgers University, Piscataway, NJ 08854 USA; 2 Department of Ecology and Evolutionary Biology, University of Arizona, Tucson, 85721 USA; 3 The Santa Fe Institute, Santa Fe, 87501 USA; 4 Department of Geography, University of California, Santa Barbara, CA 93106 USA; 5 Department of Ecology and Evolutionary Biology and Eversource Energy Center, University of Connecticut, Storrs, CT USA; 6 Moore Center for Science, Conservation International, Arlington, VA USA; 7 Department of Integrative Biology, University of South Florida, St. Petersburg, FL 33701 USA

**Keywords:** Hydrology, Natural hazards, Projection and prediction

## Abstract

Droughts are a natural hazard of growing concern as they are projected to increase in frequency and severity for many regions of the world. The identification of droughts and their future characteristics is essential to building an understanding of the geography and magnitude of potential drought change trajectories, which in turn is critical information to manage drought resilience across multiple sectors and disciplines. Adding to this effort, we developed a dataset of global historical and projected future drought indices over the 1980–2100 period based on downscaled CMIP6 models across multiple shared socioeconomic pathways (SSP). The dataset is composed of two indices: the Standardized Precipitation Index (SPI) and Standardized Precipitation Evapotranspiration Index (SPEI) for 23 downscaled global climate models (GCMs) (0.25-degree resolution), including historical (1980–2014) and future projections (2015–2100) under four climate scenarios: SSP1-2.6, SSP2-4.5, SSP3-7.0, and SSP5-8.5. The drought indices were calculated for 3-, 6- and 12-month accumulation timescales and are available as gridded spatial datasets in a regular latitude-longitude format at monthly time resolution.

## Background & Summary

Drought is a natural phenomenon that can occur in any geography and result in profound impacts on both human and natural systems across the planet. Although generally linked to anomalous lack of moisture relative to long term climatology, there is no single definition of drought due to the many ways in which deviation from average climate can be assessed, with many definitions of drought being described according to the affected domain (e.g., meteorological, agricultural, hydrological, or ecological droughts^1–5^). While droughts are part of the natural variability of the water cycle, they are especially harmful in regions of scarce water resources, rainfed agricultural lands, and fire-prone areas^6–9^. Drought-related hazards are a rising concern as future projections from global climate models indicate increasing trends in drought frequency and intensity for many areas of the world by the end of the century^10–17^.

Drought impacts are not constrained to water resources, as they can trigger other cascading hazards harmful to biodiversity and ecosystem functioning. Droughts often led to an increased risk of wildfires, with severity often directly related to drought conditions as the low moisture transforms fire-resistant green vegetation into flammable fuel^18–21^. The link between drought and vegetation dynamics has been extensively studied, with droughts influencing tree mortality, growth resilience, net primary productivity, and threatening biodiversity in different regions^22–29^. The occurrence of droughts has been linked to a broad and diverse range of impacts, such as reduced reproduction in songbirds^30^ and respiratory health issues in affected human populations^31^. Given the significant impacts of drought on livelihoods and ecosystems, considerable attention has been paid to building drought resilience through international policy such as the United Nations Convention to Combat Desertification (UNCCD). At the Conference of Parties to the UNCCD in Abidjan in 2022, Parties agreed to the need for better information on drought to inform national policy making^32^.

Identifying and characterizing drought events is typically accomplished through indices based on climate variables (e.g., precipitation, temperature, wind speed, and soil moisture). More than 100 indicators have been proposed in the literature that characterize drought type and severity with varied complexity^33^. Among them, the most utilized indicators are the Standardized Precipitation Index (SPI)^34^, Standardized Precipitation Evapotranspiration Index (SPEI)^35^, Palmer Drought Severity Index (PDSI)^36^, as well as relative statistical metrics such as percent of normal or deciles^33^. SPI and SPEI calculated over a 12-month historical period are commonly used by international policy frameworks addressing drought, such as those produced to support UNCCD Strategic Objective 3^32^.

Previous studies have provided datasets of different drought indices, for different time periods, to enable the analysis of drought impacts on both ecology and human well-being. For example, global SPEI indices at 0.5° resolution from 1901 to 2006 were introduced in the SPEIbase dataset^37^. High resolution global SPEI (5 km, ~0.05°) from 1981–2022 was produced to allow regional adaptation measures^38^. A global drought dataset based on soil moisture anomalies with a resolution of 0.25° from 1948 to 2010 was developed to incorporate snow dynamics in large-scale drought events^39^. Meteorological drought events were cataloged globally from 1951 to 2016 to provide a basis for identification of drought hotspots^40^. The Global Precipitation Climatology Centre Drought Index (GPCC-DI) provides a drought index modified from SPI and SPEI at 1° resolution from January 2013 to present^41^. Other studies have provided high resolution drought indices for Central Asia^42^, China^43^, Spain^44^, and pan-Africa^45^, allowing the assessment of localized drought impacts. Global multiscale SPEI was produced for three time periods (1960–1999, 2040–2079, and 2060–2099) based on the Fast Track experiment of the Inter-Sectoral Impact Model Intercomparison Project under representative concentration pathways (RCPs) 4.5 and 8.5 and on one Earth System Model (CMCC-CESM) forced by RCP 8.5^46^. These datasets provide useful information on droughts characteristics, however, there is no product available, to the best of our knowledge, that allows the analysis of changes in drought characteristics toward the end of the century accounting for multi-model uncertainties, different future scenarios, multiple time accumulation scales, and based on the state-of-the-art projections from CMIP6.

To fill this gap, we have developed global drought layers based on the NASA Earth Exchange Global Daily Downscaled Projections (NEX-GDDP-CMIP6) dataset^47^, with a spatial resolution of 0.25° (~25 km) and monthly time resolution, covering the period from 1980–2100. The dataset includes SPI and SPEI for 23 GCMs, including historical data (1980–2014) and future projections (2015 to 2100) for four scenarios based on the Shared Socioeconomic Pathways (SSPs): SSP1-2.6, SSP2-4.5, SSP3-7.0 and SSP5-8.5. The drought indices were calculated for 3-, 6- and 12-month timescales.

## Methods

### Reference datasets

#### Climate variables

The drought layers developed in this study are based on the NEX-GDDP-CMIP6 dataset^47^. This dataset consists of daily downscaled outputs of the Climate Model Intercomparison Project Phase 6 (CMIP6)^48^ including historical and future projections spanning from 1950 to 2100. We constrained the historical simulations to the period 1980–2014, following recommendations to minimize errors in drought assessment^49^. The outputs from NEX-GDDP-CMIP6 dataset consist of downscaled climate variables using a variation of the monthly bias correction/spatial disaggregation (BCSD) adjusted for daily data^47^. The spatial resolution of the gridded data is 0.25° (~25 km at the equator). Altogether, 23 Global Circulation Models (GCMs) were included in this analysis (Table 1) based on the availability of outputs for historic and four future projections (SSP1-2.6, SSP2-4.5, SSP3-7.0 and SSP5-8.5). The future projections reflect alternative scenarios of greenhouse gas emissions and land use, designed to assess climate change impacts under different forcing conditions^50^.

**Table 1:** Global Circulation Models (GCMs) obtained from the NEX-GDDP-CMIP6 dataset.

Model	Variant
ACCESS-CM2	r1i1p1f1
ACCESS-ESM1-5	r1i1p1f1
CanESM5	r1i1p1f1
CMCC-ESM2	r1i1p1f1
CNRM-CM6-1	r1i1p1f2
CNRM-ESM2-1	r1i1p1f2
EC-Earth3	r1i1p1f1
EC-Earth3-Veg-LR	r1i1p1f1
FGOALS-g3	r1i1p1f1
GFDL-ESM4	r1i1p1f1
GISS-E2-1-G	r1i1p1f2
INM-CM4-8	r1i1p1f1
INM-CM5-0	r1i1p1f1
IPSL-CM6A-LR	r1i1p1f1
KACE-1-0-G	r1i1p1f1
MIROC-ES2L	r1i1p1f2
MPI-ESM1-2-HR	r1i1p1f1
MPI-ESM1-2-LR	r1i1p1f1
MRI-ESM2-0	r1i1p1f1
NorESM2-LM	r1i1p1f1
NorESM2-MM	r1i1p1f1
TaiESM1	r1i1p1f1
UKESM1-0-LL	r1i1p1f2

All Models include Historical, SSP1-2.6, SSP2-4.5, SSP3-7.0 and SSP5-8.5 scenarios.

Variant identification stands for realization (r), initialization (i), physics (p) and forcing (f), followed by the identification number. For a complete description refer to CMIP6 project overview^48,65^.

The variables obtained from the NEX-GDDP-CMIP6 dataset consist of factors that can summarize an atmospheric water budget, represented by precipitation (*pr*) (Table 2), and the variables utilized to estimate potential evapotranspiration (*hurs*, *rsds*, *sfcWind*, *tas*, *tasmax* and *tasmin*) described in the section below.

**Table 2:** Description of the climate variables obtained from NEX-GDDP-CMIP6 dataset.

Variable	Description	Units
hurs	Near-surface relative humidity	percentage
pr	Precipitation (including both liquid and solid phases)	kg/m^2^/s
rsds	Surface Downwelling Shortwave Radiation	W/m^2^
sfcWind	Surface wind speed	m/s
tas	Near-surface air temperature	K
tasmax	Maximum near-surface air temperature	K
tasmin	Minimum near-surface air temperature	K

#### Global topography

We utilized a representation of the global topography at 0.25° (~25 km at the equator) based on a global 1-km topography dataset^51^. The 1-km dataset was converted to 25-km using average resampling based on the weighted average of all valid contributing pixels and reprojected to match the latitude-longitude grid of the climatic data outputs from the NEX-GDDP-CMIP6 dataset. The elevation was used to calculate the Psychrometric constant described in the next steps.

### Potential evapotranspiration (PET)

Potential evapotranspiration (PET) is a representation of the atmospheric water demand, defined as the amount of water transferred to the atmosphere through evapotranspiration in a scenario of water abundance (i.e., water availability is not a limiting factor)^52,53^. We estimated PET through the reference evapotranspiration rate (ET_0_) based on the Penman-Monteith (PM) equation, as suggested by the UN Food and Agriculture Organization (FAO) and the Environmental and Water Resources Institute of the American Society of Civil Engineering (ASCE-EWRI)^54–56^. The PM equation is defined in Eq. 1:

1 $${{\rm{ET}}}_{0}=\frac{0.408\varDelta ({R}_{n}-G)+\gamma \frac{{C}_{n}}{T+273}{u}_{2}({e}_{s}-{e}_{a})}{\varDelta +\gamma (1+{C}_{d}{u}_{2})}$$

Where:

ET_0_: reference evapotranspiration (mm day^−1^).

Δ: slope of the vapor pressure curve (kPa °C^−1^).

R_n_: net radiation at the crop surface (MJ m^−2^d^−1^).

G: soil heat flux density (MJ m^−2^d^−1^).

T: mean daily air temperature at 2 m height (°C).

u_2_: wind speed at 2 m height (m s^−1^).

e_s_: mean saturation vapor pressure (kPa).

e_a_: actual vapor pressure (kPa).

*γ*: psychrometric constant (kPa °C^−1^).

C_n_: numerator constant for the reference crop and time step.

C_d_: denominator constant for the reference crop and time step.

Following FAO-56 simplifications (i.e., reference crop with a height of 0.12 m, fixed surface resistance of 70 s m^−1^, and albedo value of 0.23), we selected a C_n_ value of 900 and C_d_ equal to 0.34. The mean daily air temperature at 2 m height (T) is an output of the downscaled GCMs, represented by the variable *tas* (Table 2) after conversion from K to Celsius. The soil heat flux density was assumed to be negligible (G = 0). PET was calculated initially at a daily scale and then converted to monthly for the drought indices calculations described in the “Drought Indices” section.

#### Wind speed at 2-meter height

The wind speed output (sfcWind, Table 2) from the GCMs is estimated at 10 m^57^. Therefore, we adjusted its value using Eq. 2^56^ for z equal to 10 m:

2 $${u}_{2}={u}_{z}\frac{4.87}{\mathrm{ln}(67.8z-5.42)}$$

Where:

u_2_: wind speed at 2 m height (m s^−1^).

u_z_: wind speed at z height (m s^−1^).

z: height of wind speed measurement above ground surface (m).

#### Slope of the vapor pressure curve (*Δ*)

The slope of the vapor pressure curve is a function of mean temperature and can be estimated by Eq. 3:

3 $$\varDelta =\frac{4098\,[0.6108{e}^{\left(\frac{17.27T}{T+237.3}\right)}]\,}{{(T+237.3)}^{2}}$$

Where:

*Δ*: slope of the vapor pressure curve (kPa °C^−1^).

T: mean daily air temperature at 2 m height (°C).

#### Psychrometric constant (*γ*)

The psychrometric constant can be described as the ratio of specific heat of moist air at constant pressure to latent heat of vaporization^56^. It can be estimated as a function of atmospheric pressure (and therefore altitude), using Eqs. 4 and 5:

4 $$\gamma =6.65\left({10}^{-4}\right)P$$

5 $$P=101.3{\left[\frac{293-0.0065z}{293}\right]}^{5.26}$$

Where:

*γ*: psychrometric constant (kPa °C^−1^).

P: atmospheric pressure (kPa).

z: elevation above sea level (m).

Elevation (z) was obtained from the topographic data described in the “Global Topography” section.

#### Mean Saturation Vapor Pressure (*e*_*s*_)

The saturation vapor pressure can be calculated as a function of air temperature (Eq. 6). The mean saturation vapor pressure can be estimated from the mean of the saturation vapor pressure at daily minimum and maximum temperature (Eq. 7).

6 $${e}_{(T)}=0.610{e}^{\left(\frac{17.27T}{T+237.3}\right)}$$

7 $${e}_{s}=\frac{{e}_{\left({T}_{\max }\right)}+{e}_{\left({T}_{\min }\right)}}{2}$$

Where:

e_s_: mean saturation vapor pressure (kPa).

T_max_: maximum daily air temperature (°C).

T_min_: minimum daily air temperature (°C).

The minimum and maximum daily temperatures are outputs from the GCMs (*tasmax* and *tasmin*, Table 2), after conversion from K to °C.

#### Actual Vapor Pressure (*e*_*a*_)

The actual vapor pressure can be calculated as a function of the relative humidity (Eq. 8):

8 $${e}_{a}=\left(\frac{{{RH}}_{{mean}}}{100}\right){e}_{s}$$

Where:

e_a_: actual vapor pressure (kPa).

e_s_: mean saturation vapor pressure (kPa).

RH_mean_: mean relative humidity (%).

The mean relative humidity is an output from the GCMs (*hurs*, Table 2). In very few instances, the near surface relative humidity (*hurs*) reported values greater than 100% in the NEX-GDDP-CMIP6 dataset. In these cases, we set relative humidity to 100%.

#### Net Radiation (*R*_*n*_)

Based on the energy budget concept, the net radiation can be calculated as the difference between the net incoming shortwave radiation and the net outgoing longwave radiation. Equations 9 to 16 can be used to estimate R_n_:

9 $${R}_{n}={R}_{{ns}}-{R}_{{nl}}$$

10 $${R}_{{ns}}=(1-\alpha ){R}_{s}$$

11 $${R}_{{nl}}=\sigma \left[\frac{{\left({T}_{\max }+273.16\right)}^{4}+{\left({T}_{\min }+273.16\right)}^{4}}{2}\right]\left(0.34-0.14\sqrt{{e}_{a}}\right)\left(1.35\frac{{R}_{s}}{{R}_{{so}}}-0.35\right)$$

12 $${R}_{{so}}=\left(0.75+2\left({10}^{-5}\right)z\right){R}_{a}$$

13 $${R}_{a}=\frac{24(60)}{\pi }{G}_{{sc}}{d}_{r}\left\{\left[{w}_{s}\cdot \sin (\varphi )\cdot \sin (\delta )\right]+\left[\sin \left({w}_{s}\right)\cdot \cos (\varphi )\cdot \cos (\delta )\right]\right\}$$

14 $${w}_{s}={arcos}\left[-\tan (\varphi )\cdot \tan (\delta )\right]$$

15 $${d}_{r}=1+0.033\cos \left(\frac{2\pi J}{365}\right)$$

16 $$\delta =0.409\sin \left(\frac{2\pi J}{365}-1.39\right)$$

Where:

*d*_*r*_: inverse relative distance Earth-Sun.

J: Julian day, i.e., number of the day in the year between 1 (1 January) and 365 or 366 (31 December).

*δ*: solar declination (rad).

*φ*: latitude (rad).

*w*_*s*_: sunset hour angle (rad).

*G*_*sc*_: solar constant (0.0820 MJ m^−2^ min^−1^).

*R*_*a*_: extraterrestrial radiation (MJ m^−2^ day^−1^).

z: elevation above sea level (m).

*R*_*so*_: clear sky solar radiation (MJ m^−2^ day^−1^).

*σ*: Stefan-Boltzmann constant (4.903(10^−9^) MJ K^−4^ m^−2^ day^−1^).

e_a_: actual vapor pressure (kPa).

T_max_: maximum daily air temperature (°C).

T_min_: minimum daily air temperature (°C).

*R*_*s*_: incoming solar radiation (MJ m^−2^ day^−1^).

*α*: albedo, which is 0.23 for FAO-56 reference crop.

*R*_*ns*_: net shortwave radiation (MJ m^−2^ day^−1^).

*R*_*nl*_: net outgoing longwave radiation (MJ m^−2^ day^−1^).

*R*_*n*_: net radiation (MJ m^−2^ day^−1^).

The incoming solar radiation (R_s_) is estimated from the output of the GCMs (*rsds*, Table 2). Latitude and time information can also be found in the NEX-GDDP-CMIP6 dataset. Very few instances near the poles resulted in negative net radiation (*R*_*nl*_ > *R*_*ns*_). In these cases, the net radiation (*R*_*n*_) was set to zero.

### Drought indices

The global drought layers presented in this study consist of two widely used drought indices, the Standardized Precipitation Index (SPI)^34^ and the Standardized Precipitation Evapotranspiration Index (SPEI)^35,58^. These two indices are commonly used to identify drought events and their severity.

#### SPI

The Standardized Precipitation Index represents normalized deviations of accumulated precipitation relative to median conditions^34^. The index is widely used due to its simplicity and range of applicability. Among its main features, SPI allows the comparison of anomalously dry conditions in regions with different climatic conditions, in addition to its ability to measure wet conditions similarly.

The procedure for calculating SPI consists of the following sequence: monthly precipitation time series for *n* years, ideally at least 30, is obtained for a given pixel or station^34^. Then, an accumulation time scale of *i*-months is defined, over which the precipitation of a given month and the *i* – 1 months preceding it is accumulated. The accumulated precipitation is a moving window within the time series, as a new value is determined from the previous months. After the accumulation, monthly subsets (totaling 12) of length *n* are drawn, one value per year of the same month. Each subset is fitted to a probability distribution, relating probability and precipitation. The original formulation suggested to fit the data to a Gamma distribution, however fitting can be problematic in regions with high occurrence of zeros, so the following function^59^ is adopted in this study:

17 $$P(x)={P}_{0}+\left({1-P}_{0}\right)\cdot F\left({x}_{x > 0},\lambda \right)$$

Where:

P: cumulative distribution function.

*P*_*0*_: frequency of zeros.

*F*(*x*_*x*>0_, *λ*): Gamma cumulative distribution function.

*λ*: parameter of gamma distribution.

x: accumulated precipitation.

Once the probability function was fitted, using the maximum likelihood estimation method, we calculated the cumulative probability (*P*) for all accumulated precipitation values in each subset. Next, we applied *P* in an inverse normal function to have normally distributed deviations with a mean of zero and a standard deviation of one. These deviations are the SPI values.

To illustrate the procedure above, consider a hypothetical time series of precipitation from January 1980 to December 2009 (*n* = 30 years). To determine the SPI value during March 2005 for a time scale of 3 months, we first accumulate the precipitation from January to March (3 months) for all years in the timeseries. This results in a subset of 30, 3-month accumulated precipitation values for Jan-Mar. This subset is then used to fit the distribution in Eq. (17). We calculate the cumulative probability using the accumulated precipitation for Jan-Mar of 2005 in the fitted distribution. Then, this cumulative probability is transformed, using an inverse normal distribution of mean zero and standard deviation one, to derive the SPI value of 3-month time scale (often referred as SPI-3) for March 2005.

We calculated SPI for 3-, 6- and 12-month time scales and constrained values to the range [−5, 5]. While a [−3, 3] range has been suggested for SPI^59^, we used [−5, 5] to accommodate regions with significant change in precipitation extremes toward the end of the century. It is worth noting that for fitting the probability distribution (Eq. 17) we used only the historic data (1980–2014). The SPI values for the future projections (SSP1-2.6, SSP2-4.5, SSP3-7.0, and SSP4-8.5 for 2015–2100) were derived by mapping the accumulated precipitation to the historical distributions. The reason for mapping future projections to historic conditions is because we aim to evaluate the future climate changes with respect to historically “normal” conditions.

#### SPEI

The Standardized Precipitation Evapotranspiration Index (SPEI^35^, incorporates the effects of temperature, and consequently global warming, in drought analysis. The index is similar to SPI, however it represents normalized deviations of the water balance instead of precipitation alone. The water balance is represented by the difference between precipitation and potential evapotranspiration (PET, Eq. 1) at a monthly scale.

The SPEI calculation is also similar to the SPI, however the time series consists of precipitation minus PET. Also, the monthly subsets of accumulated precipitation - PET values are fitted to a Generalized Extreme Value (GEV) instead of using Eq. 17. The original framework^35^ proposed a 3-parameter log-logistic distribution, however the log-logistic function does not have a real solution for some combinations of precipitation and PET^59^, so the GEV framework was adopted here. Parameter estimation was based on the maximum likelihood estimation method in this case as well. We calculated SPEI for 3-, 6- and 12-month time scales, constraining the values to the range [−5, 5]. The GEV function was fitted using the historic data (1980–2014), and future projections were mapped to this distribution, following the same rationale described in the SPI methodology.

## Data Records

The Global Drought Layers presented in this study are composed of SPI and SPEI indices for 23 GCMs (Table 1) of NEX-GDDP-CMIP6 dataset, including historical (1980–2014) data and future projections (2015–2100) under four climate scenarios: SSP1-2.6, SSP2-4.5, SSP3-7.0, and SSP5-8.5. The drought indices were calculated for 3-, 6- and 12-month accumulation timescales. The data are gridded in a regular latitude-longitude format, with a spatial resolution of 0.25° (~25 km at the equator) and monthly time resolution, covering the period 1980–2100. The drought layers described in this paper are freely available at the NASA Socioeconomic Data and Applications Center (SEDAC) (10.7927/4es0-1v73)^60^ through a Creative Commons Attribution 4.0 International License. The files are in GeoTIFF format separated by date, GCM, scenario, and time scale. Each file size is ~1.4 MB, and the total archive is ~800GB.

## Technical Validation

**Fig. 1:**
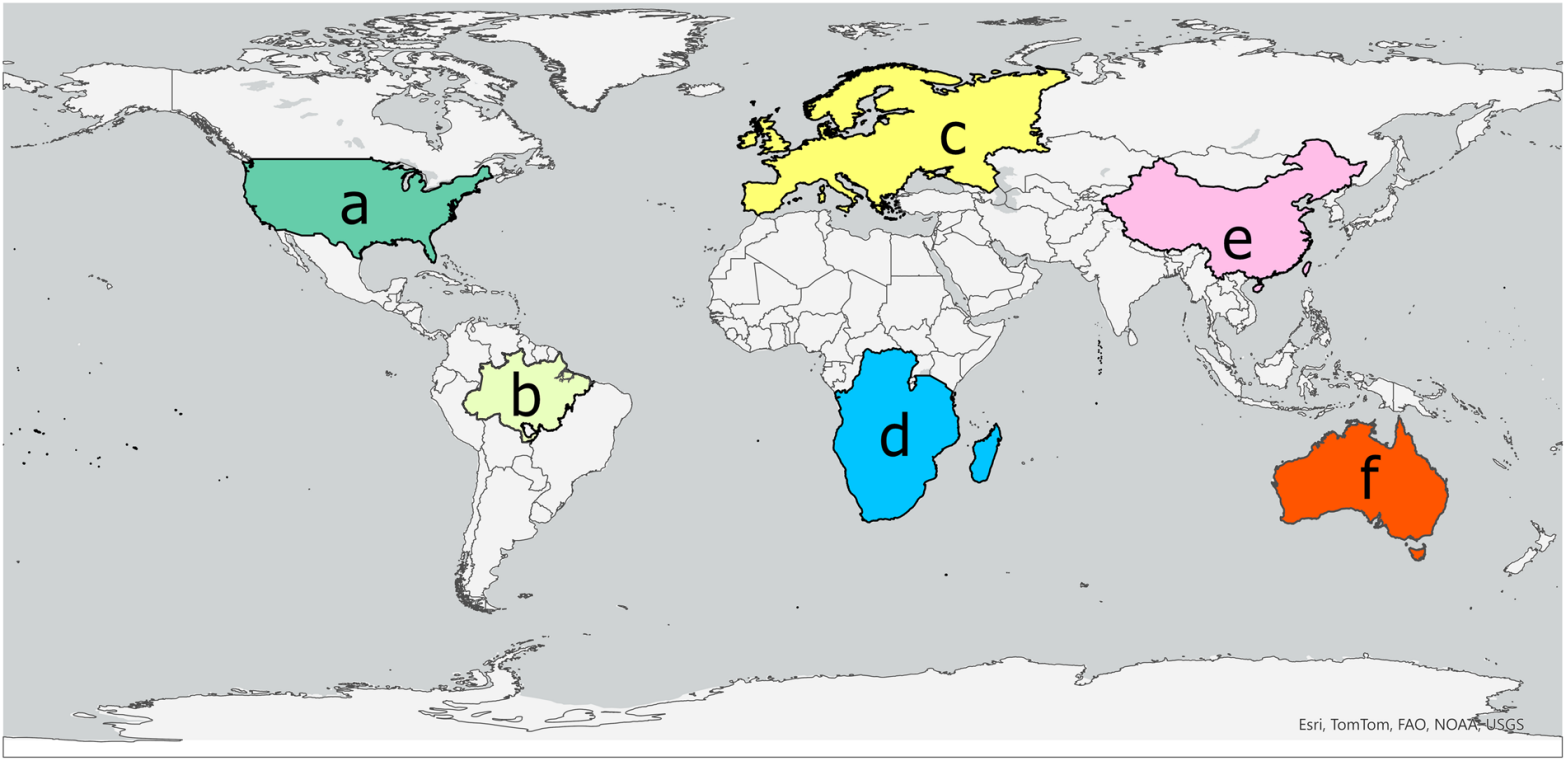
Representation of the six regions of interest, (**a**) contiguous United States (CONUS), (**b**) the Amazon, (**c**) Europe, (**d**) Southern Africa, including Angola, Tanzania, Congo DRC, Zambia, Mozambique, Namibia, Botswana, Zimbabwe, South Africa and Madagascar, (**e**) China and (**f**) Australia.

The methods used to create this dataset, including calculating Penman-Monteith PET, SPEI, and SPI, have been extensively tested and validated in other studies^34,35,55,59,61–63^. To demonstrate the validity of our datasets, we compared the drought characteristics (duration, severity and number of events) to ones derived from ERA5-Land^64^. The ERA5-Land dataset provides monthly precipitation and potential evaporation at ~9 km resolution, through which we calculated SPEI for the 1980–2014 period and 3-, 6- and 12-month time scale. This period was selected to match the historic part of our dataset. The SPEI index was selected because it allows for the comparison of precipitation and PET effects combined. We selected six regions to represent different areas of the planet in each continent (Fig. 1).

**Fig. 2:**
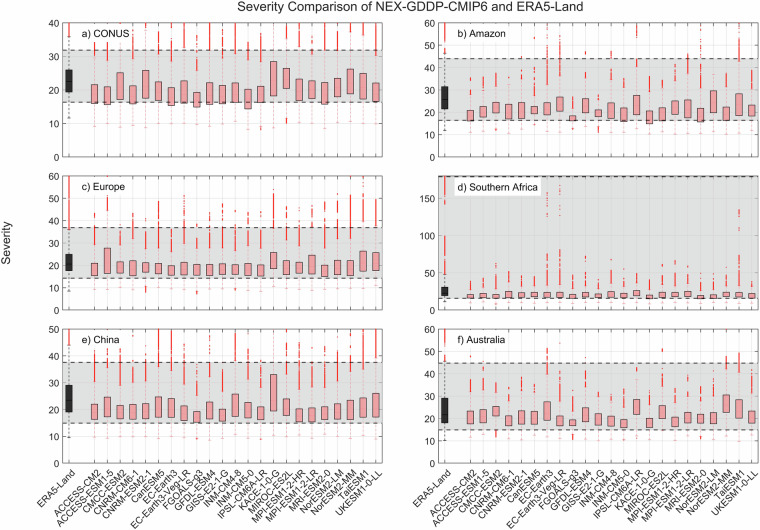
Comparison of mean severity between the drought indices (SPEI-12) derived through NEX-GDDP-CMIP6 (red boxes) and ERA5-Land (black box) for: (**a**) Contiguous United States (CONUS), (**b**) the Amazon, (**c**) Europe, (**d**) Southern Africa, (**e**) China and (**f**) Australia. The gray area corresponds to the boundaries of the fifth and 95^th^ percentile of the ERA-Land values.

**Fig. 3:**
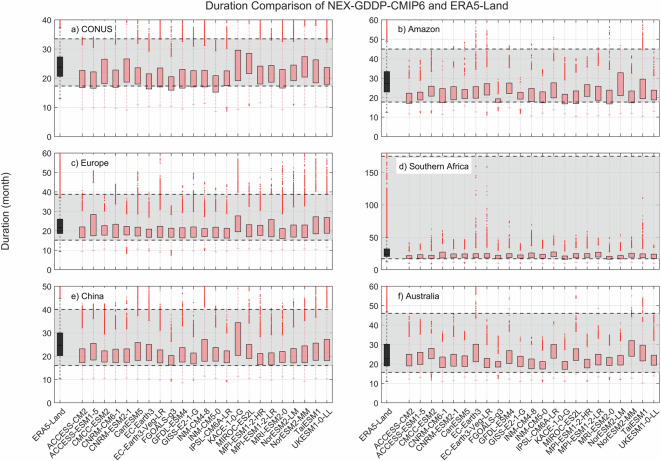
Comparison of mean duration (months) between the drought indices (SPEI-12) derived through NEX-GDDP-CMIP6 (red boxes) and ERA5-Land (black box) for: (**a**) Contiguous United States (CONUS), (**b**) the Amazon, (**c**) Europe, (**d**) Southern Africa, (**e**) China and (**f**) Australia. The gray area corresponds to the boundaries of the fifth and 95^th^ percentile of the ERA-Land values.

**Fig. 4:**
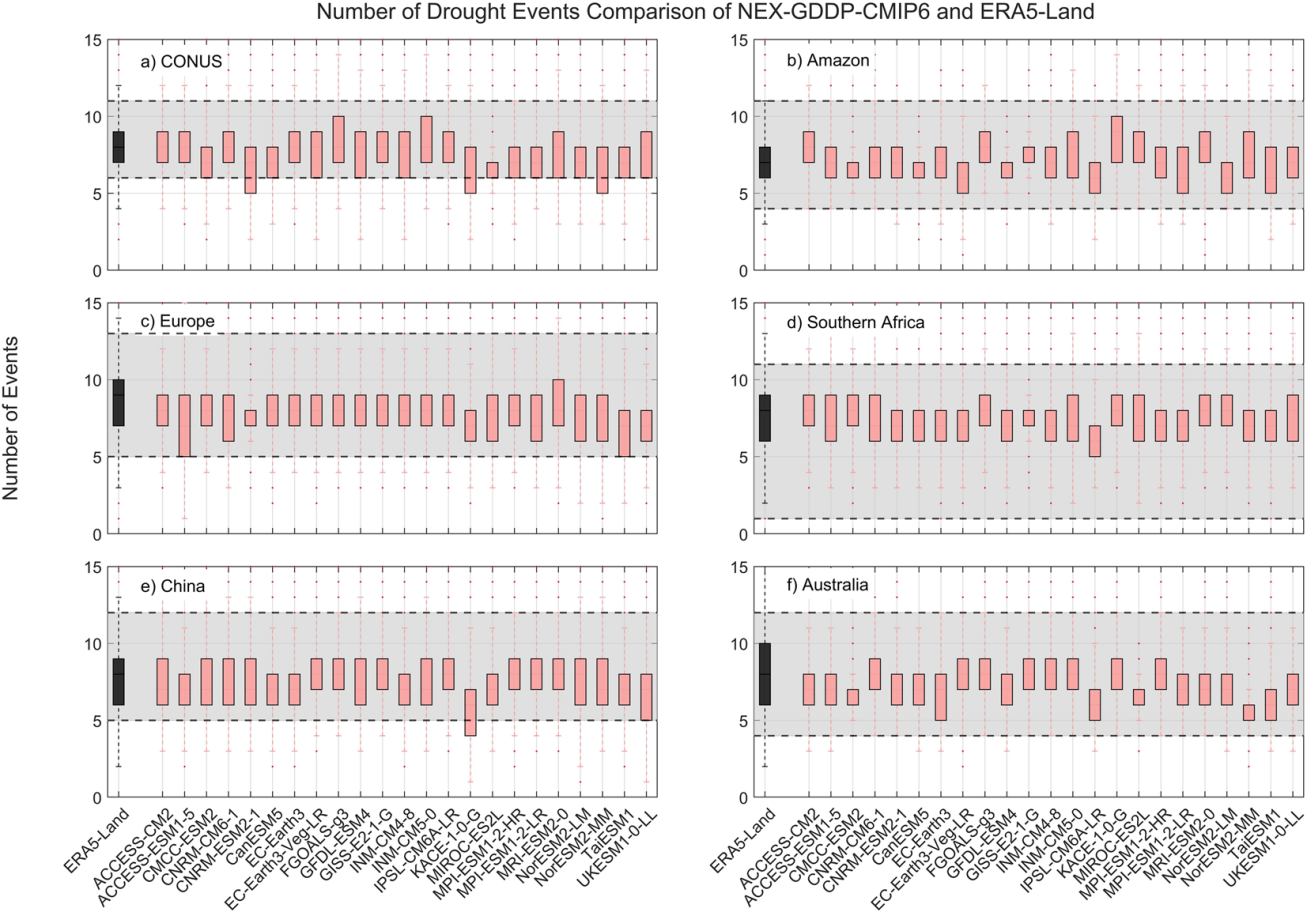
Comparison of mean number of drought events between the drought indices (SPEI-12) derived through NEX-GDDP-CMIP6 (red boxes) and ERA5-Land (black box) for: (**a**) Contiguous United States (CONUS), (**b**) the Amazon, (**c**) Europe, (**d**) Southern Africa, (**e**) China and (**f**) Australia. The gray area corresponds to the boundaries of the fifth and 95^th^ percentile of the ERA-Land values.

**Fig. 5:**
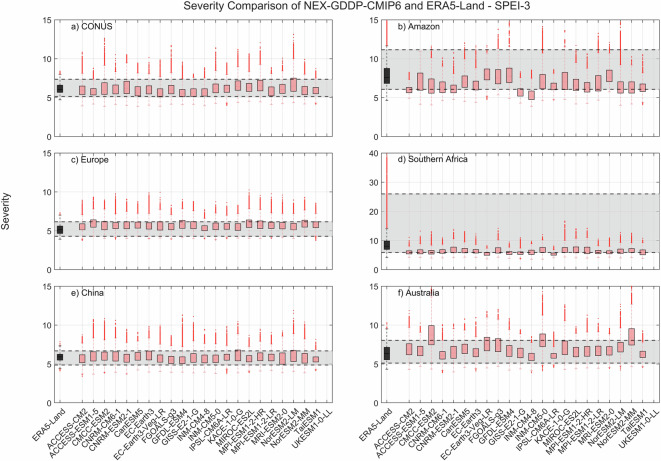
Comparison of mean severity between the drought indices (SPEI-3) derived through NEX-GDDP-CMIP6 (red boxes) and ERA5-Land (black box) for: (**a**) Contiguous United States (CONUS), (**b**) the Amazon, (**c**) Europe, (**d**) Southern Africa, (**e**) China and (**f**) Australia. The gray area corresponds to the boundaries of the fifth and 95^th^ percentile of the ERA-Land values.

**Fig. 6:**
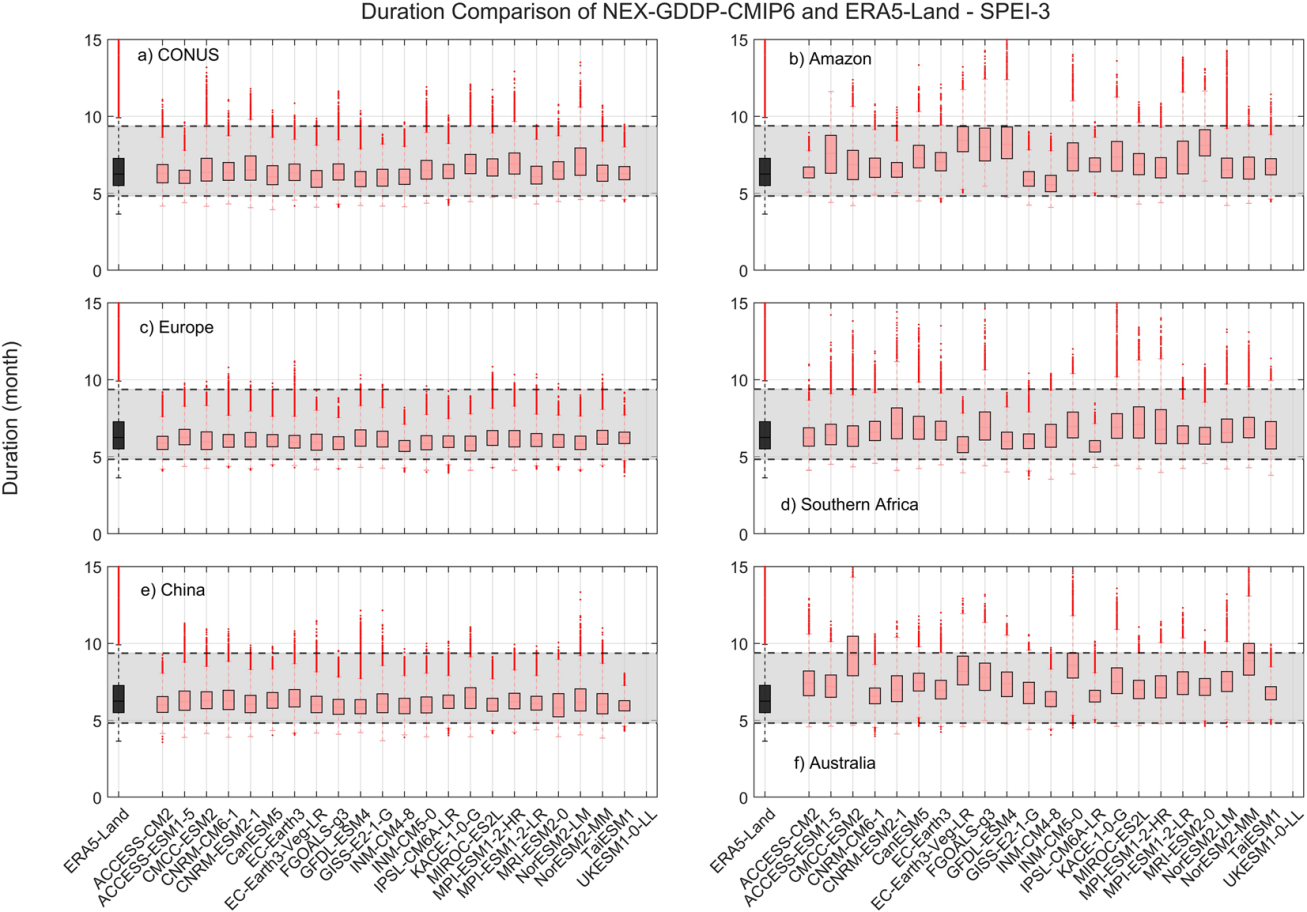
Comparison of mean duration (months) between the drought indices (SPEI-3) derived through NEX-GDDP-CMIP6 (red boxes) and ERA5-Land (black box) for: (**a**) Contiguous United States (CONUS), (**b**) the Amazon, (**c**) Europe, (**d**) Southern Africa, (**e**) China and (**f**) Australia. The gray area corresponds to the boundaries of the fifth and 95^th^ percentile of the ERA-Land values.

**Fig. 7:**
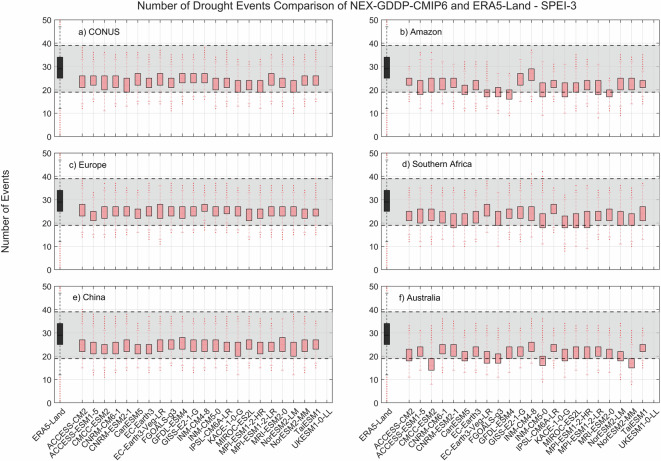
Comparison of mean number of drought events between the drought indices (SPEI-3) derived through NEX-GDDP-CMIP6 (red boxes) and ERA5-Land (black box) for: (**a**) Contiguous United States (CONUS), (**b**) the Amazon, (**c**) Europe, (**d**) Southern Africa, (**e**) China and (**f**) Australia. The gray area corresponds to the boundaries of the fifth and 95^th^ percentile of the ERA-Land values.

**Fig. 8:**
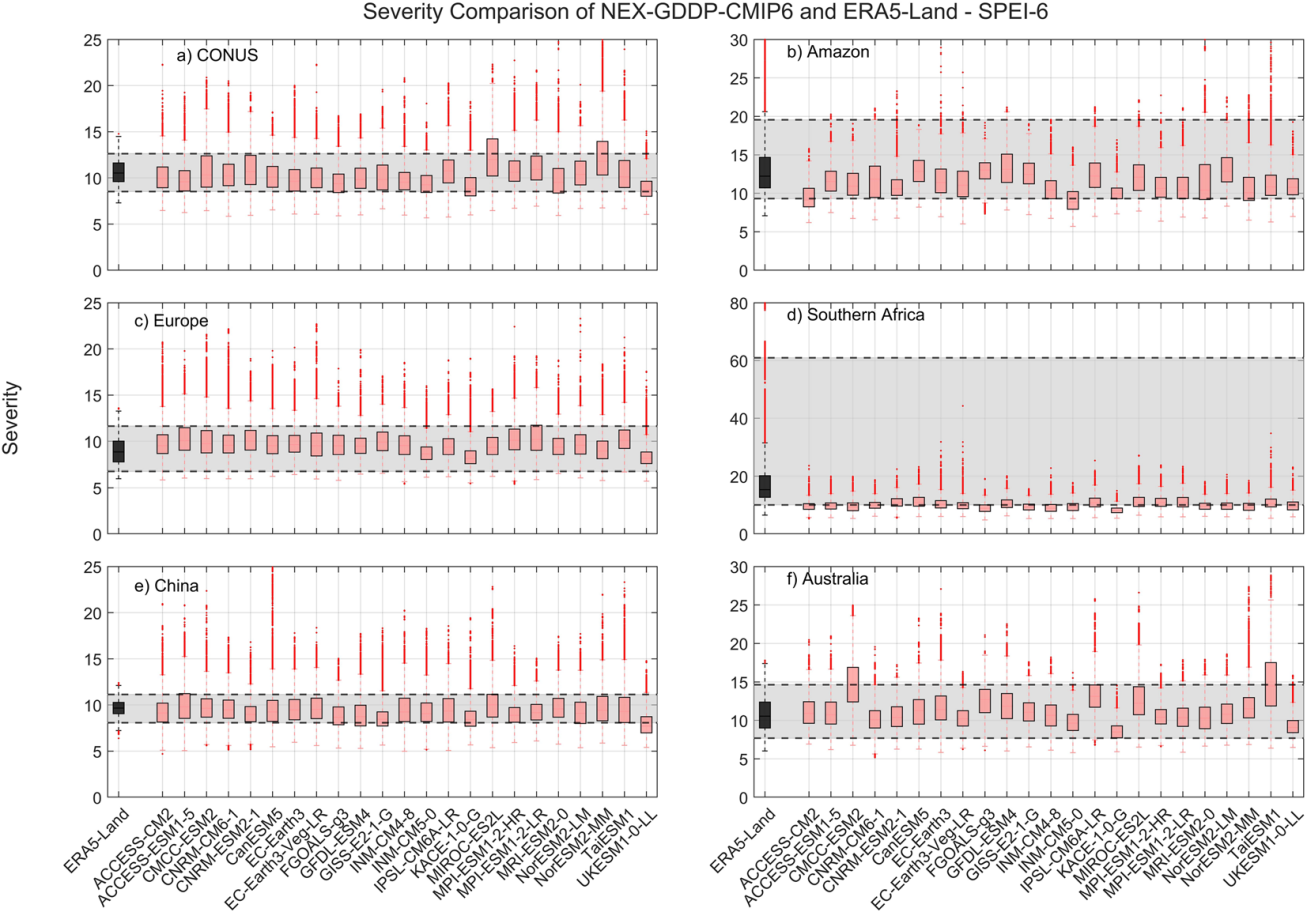
Comparison of mean severity between the drought indices (SPEI-6) derived through NEX-GDDP-CMIP6 (red boxes) and ERA5-Land (black box) for: (**a**) Contiguous United States (CONUS), (**b**) the Amazon, (**c**) Europe, (**d**) Southern Africa, (**e**) China and (**f**) Australia. The gray area corresponds to the boundaries of the fifth and 95^th^ percentile of the ERA-Land values.

**Fig. 9:**
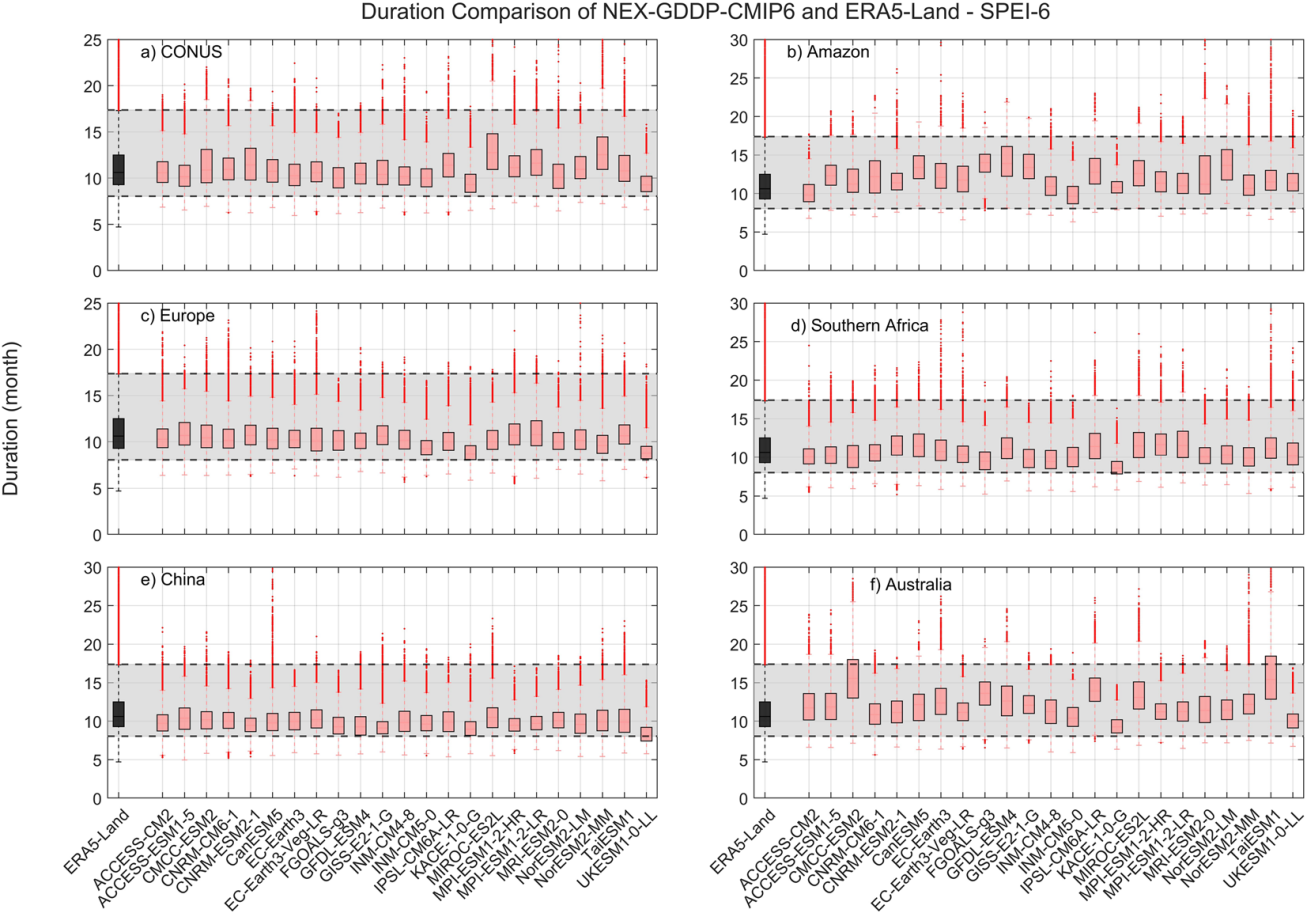
Comparison of mean duration (months) between the drought indices (SPEI-6) derived through NEX-GDDP-CMIP6 (red boxes) and ERA5-Land (black box) for: (**a**) Contiguous United States (CONUS), (**b**) the Amazon, (**c**) Europe, (**d**) Southern Africa, (**e**) China and (**f**) Australia. The gray area corresponds to the boundaries of the fifth and 95^th^ percentile of the ERA-Land values.

**Fig. 10:**
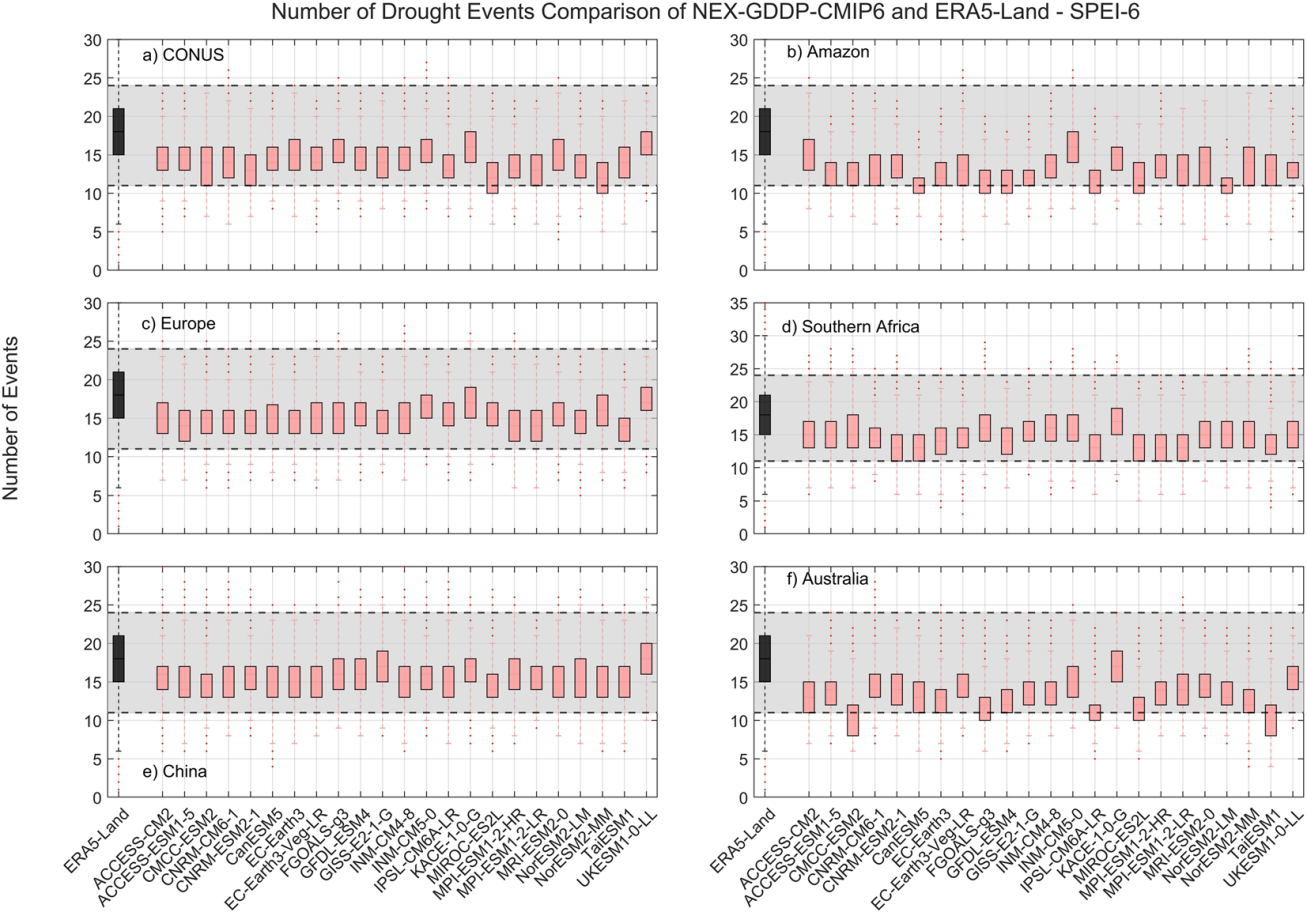
Comparison of mean number of drought events between the drought indices (SPEI-6) derived through NEX-GDDP-CMIP6 (red boxes) and ERA5-Land (black box) for: (**a**) Contiguous United States (CONUS), (**b**) the Amazon, (**c**) Europe, (**d**) Southern Africa, (**e**) China and (**f**) Australia. The gray area corresponds to the boundaries of the fifth and 95th percentile of the ERA-Land values.

The comparison metric consisted in mean duration, severity, and number of events for the period 1980–2014 for each pixel within the region of interest. The results for SPEI show that while there is variability among GCMs, as expected, the range of all drought characteristics (severity, duration and number of events) examined during the historic period are within the same range of values derived from ERA5-Land reanalysis (Figs. 2–4). A one-to-one correspondence between NEX-GDDP-CMIP6 and ERA5-Land is not expected since the GCMs are only offering a realization of historic climate and do not incorporate actual observations of historic climate, as is the case for ERA5-Land reanalysis. The results of the comparisons were consistent across the selected regions of the world. Therefore, we can consider the drought indices in our dataset to be in a realistic range and that they are able to represent temporal and spatial variability of drought conditions at global scale. Similar conclusions are obtained when considering 3- and 6-month time scales. Results for these cases are summarized in Figs. 5–10. Additional validation for specific uses of the data may be warranted depending on user intentions.

## Usage Notes

**Fig. 11:**
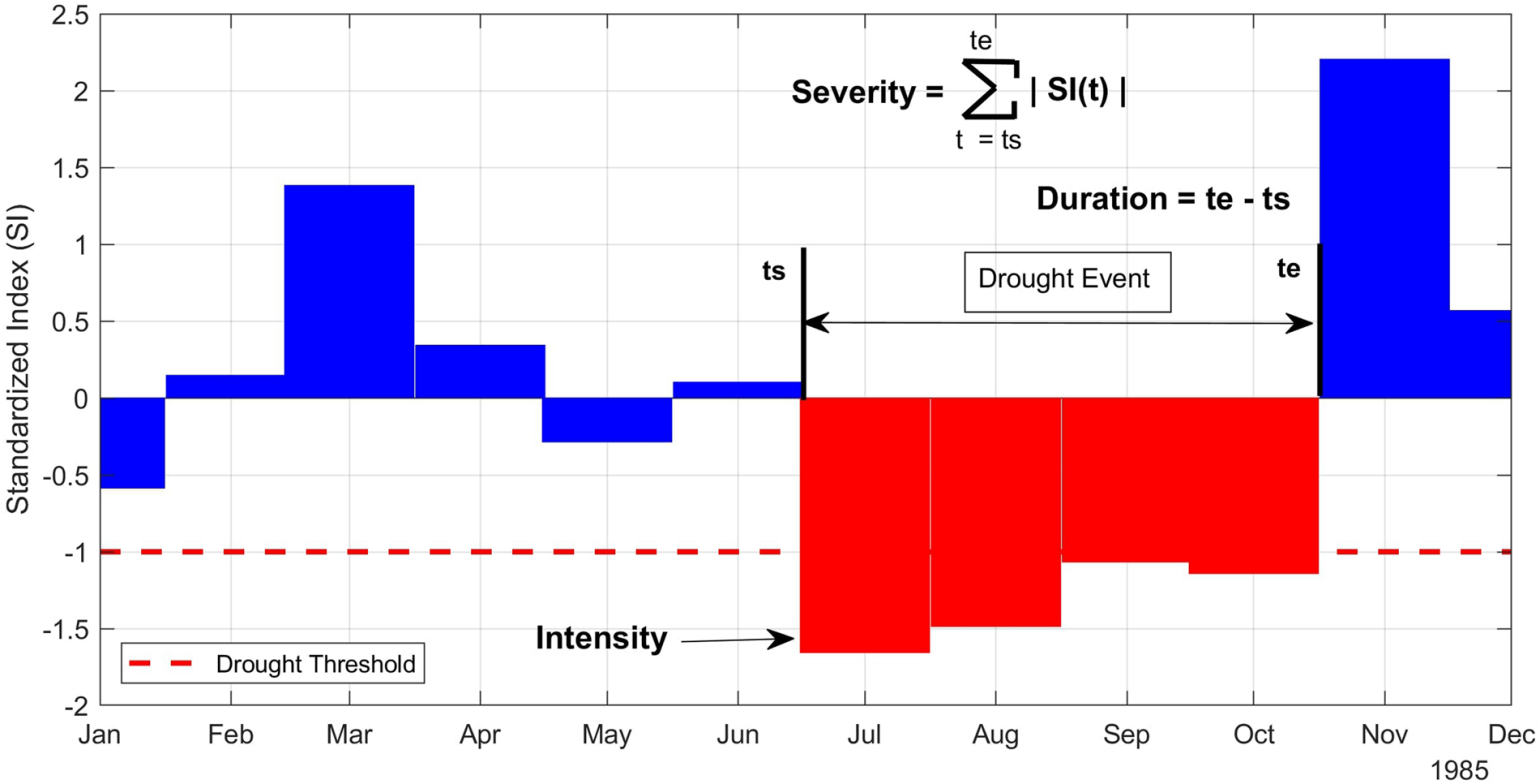
Schematic representation of drought event identification and its characteristics definition based on a hypothetical Standardized Index for the year 1985. *Ts* and *Te* stands for starting and ending time.

The standardized indices (SI; referring to both SPI and SPEI) defined above can be used to identify drought events and consequently define their characteristics. Drought events are defined as a period in which an SI is continuously negative and reaches a value of −1.0 or less^34^ (Fig. 11). The drought starts when the SI first drops below zero and ends with the first positive value of the SI succeeding a value of −1.0 or less.

Building on the definition of drought events above, we can establish three main characteristics of droughts: intensity, duration, and severity (Fig. 11). Intensity is represented by the minimum value of SI within the drought event. Droughts are commonly classified by intensity following^34^: mild if SI *ϵ*[0, −0.99], moderate if SI ϵ[−1.00, −1.49], severe if SI *ϵ*[−1.50, −1.99], or extreme if SI *ϵ*[−2.00, −5.00]. Duration is defined as the period length between the start and end of a drought event, while severity is represented by the summation of all SI values within the event.

## Data Availability

The codes used to calculate PET and the drought indices were created in MATLAB version R2020b, they can be found at https://github.com/HydroReS/Droughts.
